# Trial Promoter: A Web-Based Tool for Boosting the Promotion of Clinical Research Through Social Media

**DOI:** 10.2196/jmir.4726

**Published:** 2016-06-29

**Authors:** Katja Reuter, Francis Ukpolo, Edward Ward, Melissa L Wilson, Praveen Angyan

**Affiliations:** ^1^ Southern California Clinical and Translational Science Institute Keck School of Medicine of USC University of Southern California Los Angeles, CA United States; ^2^ Departments of Preventive Medicine & Obstetrics and Gynecology Keck School of Medicine of USC University of Southern California Los Angeles, CA United States

**Keywords:** algorithm, automation, clinical trial, communication, Facebook, Internet, online, patient, recruitment, social network, social media, Twitter

## Abstract

**Background:**

Scarce information about clinical research, in particular clinical trials, is among the top reasons why potential participants do not take part in clinical studies. Without volunteers, on the other hand, clinical research and the development of novel approaches to preventing, diagnosing, and treating disease are impossible. Promising digital options such as social media have the potential to work alongside traditional methods to boost the promotion of clinical research. However, investigators and research institutions are challenged to leverage these innovations while saving time and resources.

**Objective:**

To develop and test the efficiency of a Web-based tool that automates the generation and distribution of user-friendly social media messages about clinical trials.

**Methods:**

Trial Promoter is developed in Ruby on Rails, HTML, cascading style sheet (CSS), and JavaScript. In order to test the tool and the correctness of the generated messages, clinical trials (n=46) were randomized into social media messages and distributed via the microblogging social media platform Twitter and the social network Facebook. The percent correct was calculated to determine the probability with which Trial Promoter generates accurate messages.

**Results:**

During a 10-week testing phase, Trial Promoter automatically generated and published 525 user-friendly social media messages on Twitter and Facebook. On average, Trial Promoter correctly used the message templates and substituted the message parameters (text, URLs, and disease hashtags) 97.7% of the time (1563/1600).

**Conclusions:**

Trial Promoter may serve as a promising tool to render clinical trial promotion more efficient while requiring limited resources. It supports the distribution of any research or other types of content. The Trial Promoter code and installation instructions are freely available online.

## Introduction

Scarce information about clinical research, in particular clinical trials, is among the top reasons why potential participants do not take part in clinical trials. Clinical trials are vital for the development of novel approaches to advancing medicine, but without volunteers this type of research is impossible. In 2012, the Institute of Medicine recognized the seriousness of the clinical trial participation problem [1] and released a report that identified numerous barriers, including the lack of awareness among patients and physicians that clinical trials are available. New solutions are needed that increase clinical trial awareness and build rapport among patients, physicians, and caregivers with the aim to boost clinical trial engagement and recruitment rates.

We have developed and tested Trial Promoter, a Web-based tool that automatically generates and distributes social media messages about clinical trials. New digital options such as social media (ie, social networks) have the potential to work alongside traditional methods to boost the promotion of clinical research. Social media describe websites and Web-based applications that enable users to create and share content or to participate in social networking. With millions of users, social media serve as a promising solution to improve the public awareness of clinical trials and to support research participant recruitment efforts. In fact, the use of the Internet as a top source for clinical research information has increased significantly (46% in 2013), whereas the use of mass media has declined (newspaper, radio, television; 39% in 2013) [2]. Between 30% and 40% of the public reports that they have used social media to gather medical information to learn about clinical research, with the social network Facebook topping the list [3-6]. This trend is not limited to young adults; half of people aged 50 years and older and more than a third of people aged 65 years and older frequent social networking sites such as Twitter and Facebook [7-9]. These data suggest that patients, caregivers, and disease advocates can be found, informed, and engaged digitally. We have built Trial Promoter to leverage this digital trend and to support research institutions that seek to respond to the evolving way in which patients, physicians, caregivers, and advocacy groups search for, create, and use health information online.

Trial Promoter builds on preliminary work where we tested an automated approach to generate and publish messages about research-related content on Twitter. Our work indicated that a machine algorithm helps research teams and institutions to increase the output and reach of information about research on social media while reducing the burden of developing and distributing hundreds of messages [10].

Here we present the tool and preliminary data, suggesting that Trial Promoter may aid in distributing clinical trial information more broadly while requiring limited resources. The tool serves four functions: first, it imports information from specific databases or data files. Second, it generates user-friendly social media messages based on preapproved message templates. Third, it schedules and distributes these messages through the social media platforms Twitter and Facebook. Fourth, it tracks the success of the messages and displays their engagement and conversion metrics data. The source code and installation instructions are freely available online [11]. In order to test the tool, we conducted a 10-week trial. Trial Promoter randomized 46 active and recruiting clinical trials into social media messages and distributed them via Twitter and Facebook. We assessed the correctness of the test messages and calculated the probability with which Trial Promoter generated accurate messages.

## Methods

### Trial Promoter Setup

#### System Requirements

Trial Promoter is built using Ruby on Rails (version 4.2.1), HTML, cascading style sheet (CSS), and JavaScript. We have installed Trial Promoter on Ubuntu Linux 14.04 LTS (long-term support) and use Phusion Passenger, a scalable Web server that hosts Trial Promoter. Trial Promoter further uses PostgreSQL 9.4.5 database systems deployed on Ubuntu 14.04 LTS. Administrator privileges for setting up Cron jobs are required in order to set up nightly data extractions that secure logs, collate metrics, and distribute messages.

#### Information Import

**Figure 1:**
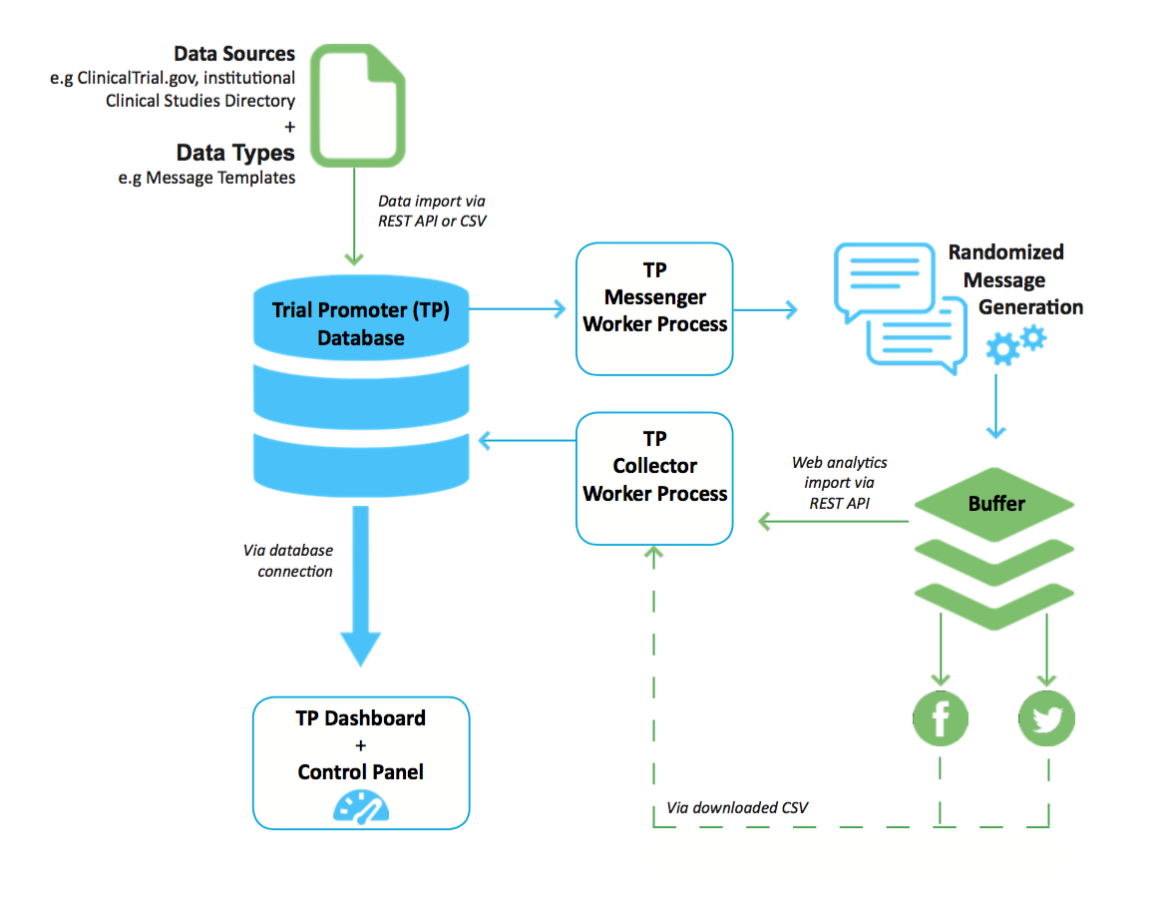
Trial Promoter (TP) setup and data flow. The elements in blue represent functional TP modules. CSV: comma-separated values; REST API: representational state transfer application programming interface.

**Figure 2:**
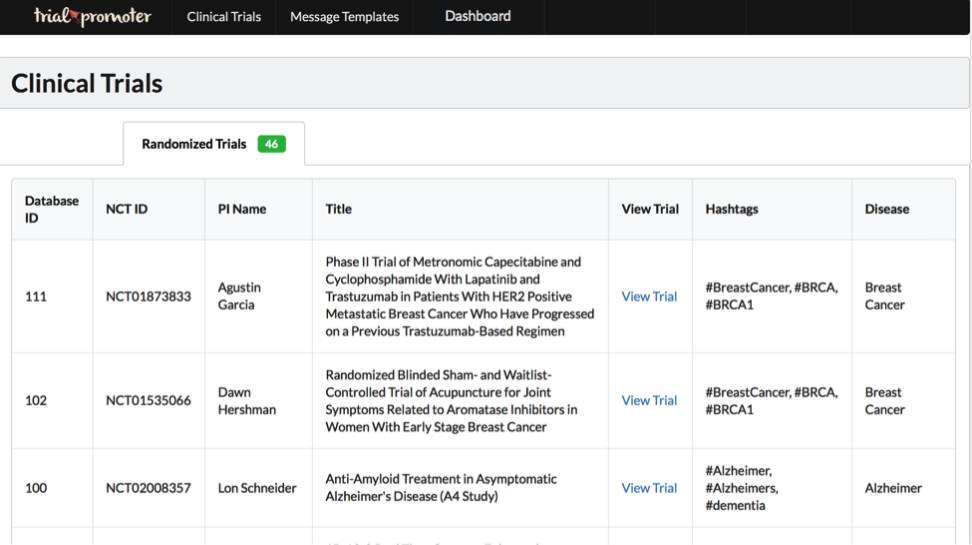
Local Trial Promoter interface that shows imported clinical trial information and disease keywords that were included in the test messages.

Trial Promoter has the capability to import information from different databases and data files through either a representational state transfer (REST) application programming interface (API) or a comma-separated values (CSV) file. Figure 1 depicts the Trial Promoter setup and data flow. Figure 2 represents a screenshot of the local Trial Promoter interface that shows imported clinical trial information and disease keywords that were included in the test messages. Table 1 lists the information our local Trial Promoter installation imported for testing purposes, for example, clinical trial information from our institutional Clinical Studies Directory that utilizes data from ClinicalTrials.gov provide by the National Library of Medicine [12], message templates designed for Twitter and Facebook, and information on disease hashtags. Disease hashtags are disease keywords preceded by a pound sign (eg, #Diabetes, #BreastCancer). They are used by members of disease communities on social media sites such as Twitter to identify and discover messages on a specific topic [13,14].

**Table 1:** Data sources and types of data imported for testing purposes by our local installation of Trial Promoter.

Imported content	Data source/format	Data types
**Active, recruiting clinical trials**	Clinical Studies Directory/REST API^a^	Clinical trial title
		Name of principal investigator
		Clinical trial landing page URL
**Disease hashtags**	Symplur [11]/CSV^b^ file	Disease keywords
**Message templates**	N/A^c^/CSV file	Text message templates designed for Twitter and Facebook

^a^REST API: representational state transfer application programming interface.

^b^CSV: comma-separated values.

^c^N/A: not applicable.

#### Message Generation and Distribution

**Figure 3:**
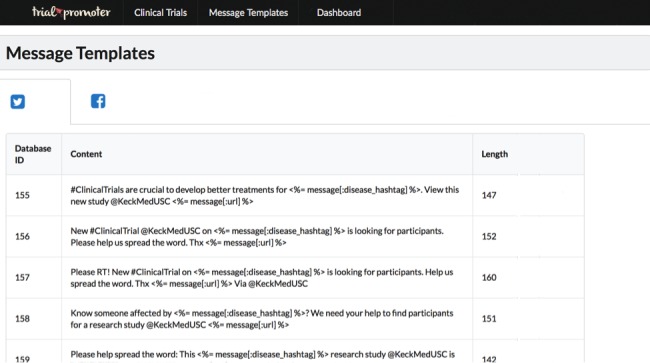
Local Trial Promoter interface shows parameterized message templates for Twitter and Facebook that were used during testing.

Trial Promoter Messenger develops social media messages tailored to Twitter and Facebook. To achieve that, Trial Promoter uses preapproved message templates that we developed with input from communication experts at Keck Medicine of the University of Southern California (USC). During testing, we used 154 parameterized message templates. Figure 3 represents a screenshot of the local Trial Promoter interface that shows parameterized message templates for Twitter and Facebook, which were used during testing. The parameterization supports the generation of large sets of messages from a limited set of clinical trials and message templates. The words in italics the following example messages represent parameters that Trial Promoter added into the message templates to create the final social media messages.

Example of Twitter message template: “New #ClinicalTrial *@KeckMedUSC* on *#disease* is looking for participants. Please help us spread the word. Thx. *URL* ”

Example of Facebook message template: “Your help is appreciated: New clinical trial at Keck Medicine of USC on *#disease* is looking for participants. Through these types of clinical studies researchers can better understand how to diagnose, treat and prevent diseases. Please share this *URL*. Thanks!”

Trial Promoter matches a randomly chosen clinical trial with a randomly chosen message template using the standard Ruby library to generate random numbers [15]. The random numbers in the library are implemented as a modified Mersenne Twister with a period of 2^19937^−1 [16]. Trial Promoter shuffles all clinical trials and then randomly chooses a message template for each trial [17], ensuring that all clinical trials are only distributed once during a given time period.

Trial Promoter then substitutes the parameters in the message templates and includes several weblinks into the message templates to create the final messages: first, a tagged and shortened URL that links the social media message to the respective clinical trial landing page; second, a primary and if applicable a secondary disease hashtag (eg, #LungCancer, #SleepApnea); and third, for Twitter messages the official Keck Medicine of USC Twitter account (@KeckMedUSC). Table 2 lists the characteristics of the messages that Trial Promoter generated automatically during the testing phase.

**Table 2:** Characteristics of test messages that Trial Promoter generated automatically for distribution on Twitter and Facebook.

Characteristic	Twitter	Facebook
Maximum message length limitation	Limitation to 140 characters^a^	N/A^b^
Parameter: URL	22 characters for non-https URLs 23 characters for https URLs	Links can be of any length. However, in order to simplify URL sharing and present clean URLs to the Facebook page visitor, Trial Promoter uses Bit.ly shortened URLs on Facebook posts as well.
Parameter: hashtags (disease keyword)	Yes (primary and if length permits secondary hashtag)	Yes (primary and secondary hashtags)
Parameter: link to official Keck Medicine of USC^c^ Twitter account (@KeckMedUSC)	Yes	N/A^b^

^a^Note: Media attachments such as photos, videos, and polls are not counted toward 140 characters.

^b^N/A: not applicable.

^c^USC: University of Southern California.

Furthermore, Trial Promoter tags the URL that links to the clinical trial landing page with Urchin Traffic Monitor (UTM) parameters in order to track the link engagement (or clicks) on social media and referral traffic to the clinical trial landing page [18]. During testing, Trial Promoter used the REST API provided by the Bit.ly link-shortening service to generate the shortened URL [19]. Bit.ly preserves the UTM parameters by mapping identical links with different UTM parameters to unique URLs.

For Twitter, the automated inclusion of disease hashtags that vary in length depending on the disease term used may result in messages that are longer than 140 characters (eg, #HIV vs #PancreaticCancer). If the generated message was greater than 140 characters, Trial Promoter discarded the message and selected an alternative message template until it either generated a valid message or it ran out of message templates to choose from. In the latter case, Trial Promoter notified the study team of the error in the administrative dashboard.

*Trial Promoter Messenger* then schedules and distributes the test messages through selected Twitter and Facebook accounts (eg, USC Clinical Trials) using the social media content management Web application Buffer [20]. Each social media account set up in Buffer has a unique profile identifier (ID) assigned to it. Buffer provides a REST API call that allows for programmatic scheduling of messages directly in Buffer. With a single call, the Buffer REST API [21] sends a single message to multiple social media channels. The REST API provides options for scheduling messages and including images. Once messages are sent to Buffer, the tool interface allows users to edit, reschedule, reorder, and delete messages.

#### Automated Tracking of Message Success

*Trial Promoter Collector* tracks key performance indicators (KPIs) associated with the success of the messages, that is, social media engagement and landing page conversion data. Table 3 lists the engagement and conversion KPIs tracked by Trial Promoter. Trial Promoter Collector extracts these data on a nightly basis from 4 existing systems: Buffer, Twitter, Facebook, and Google Analytics [22]. The tool connects to Buffer via a REST API in order to extract the social media and link engagement data for each social media message sent via Buffer. To collect the data from Twitter and Facebook, our team uploaded reports taken from each social media platform to Trial Promoter. Trial Promoter processed these raw logs and imported them into its dashboard on a nightly basis. The landing page conversion data are accessible through Google Analytics. To extract the data, Trial Promoter required uploads of the Google Analytics data report into Trial Promoter. Trial Promoter then processed and incorporated the data into its dashboard.

**Table 3:** Engagement and conversion key performance indicators tracked by Trial Promoter.

Metric categories	Measures on Twitter	Measures on Facebook
Volume of messages served	Impressions	Impressions
Social media engagement	Retweets Replies Likes	Shares Comments Likes
Link engagement	Clicks from social media message to clinical trial landing page on Clinical Studies Directory	Clicks from social media message to clinical trial landing page on Clinical Studies Directory
Landing page engagement	Sessions Time spent on page Pageviews per visit	Sessions Time spent on page Pageviews per visit
Contact engagement	Contact form usage on individual clinical trial information page	Contact form usage on individual clinical trial information page

**Figure 4:**
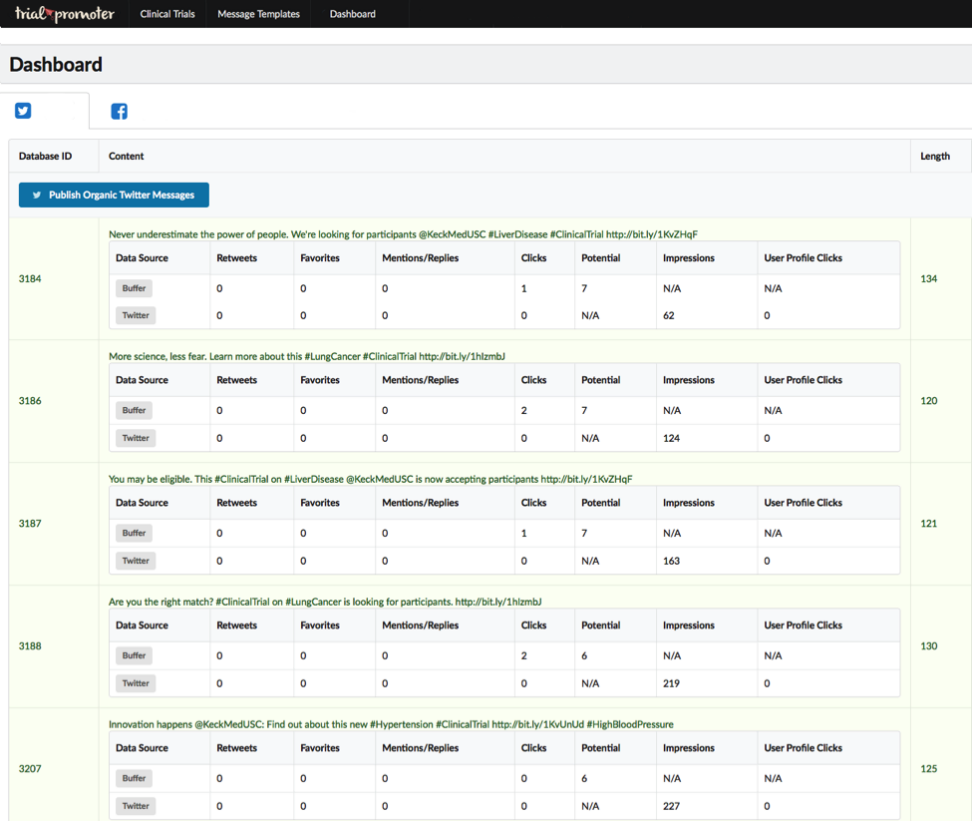
Local Trial Promoter interface shows key performance indicator data for each message. This example shows Twitter messages.

Finally, *Trial Promoter Dashboard* displays the KPI data, providing daily updates. Figure 4 represents a screenshot of the local Trial Promoter interface that shows KPI data for each Twitter message. During testing, Trial Promoter matched up data from the raw data logs for each social media platform to the data from Buffer using a unique social message (or update) ID. Each social media channel generates a unique ID for every message that is sent out via its platform. Using this unique ID allowed us to match up entries in the raw data logs to a specific message. Additionally, the Trial Promoter Dashboard serves as a control panel to add, edit, and delete clinical trials, message templates, and social media messages and images.

### Trial Promoter Evaluation

#### Test Trial Design

During the 10-week test trial, Trial Promoter randomized clinical trials (n=46) into social media messages using preapproved message templates. The tool generated, scheduled, and published 2 messages on each platform (Twitter and Facebook) per day, and 3 messages per platform every other day.

#### Correctness Analysis

The correctness with which Trial Promoter generated messages during the 10-week testing phase was evaluated using 4 indicators: (1) the correct usage of the message template, (2) the text of the message itself (ie, number of text errors in the message), (3) the inclusion of the correct URL, and (4) the inclusion of the correct disease hashtags. The individual indicators were averaged to obtain the overall percent correct. The correct usage of the message template was measured through random sampling 25/525 messages (5%) and manually comparing the message template with the social media that was generated by Trial Promoter. The correctness of the included URL was evaluated using a script written in Ruby on Rails [8]. The script examined the URL by first expanding the shortened Bit.ly URL to a complete URL. The URL was then compared with the landing page of the clinical trial being promoted using regex expressions (ignoring any query strings in the URL) to ensure that they were identical. The inclusion of the correct disease hashtags (eg, #Stroke, #LungCancer) was manually reviewed in all 525 messages upon scheduling.

## Results

### Trial Promoter Evaluation

**Figure 5:**
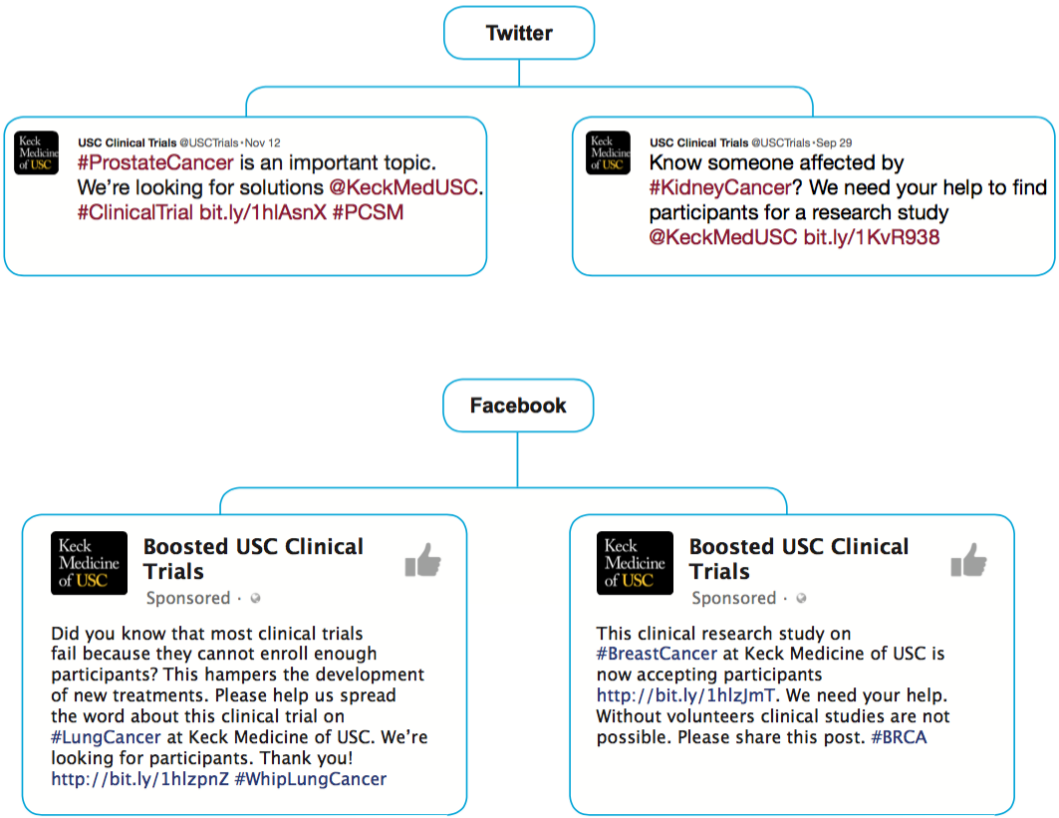
Examples of messages that Trial Promoter generated and published automatically.

During the 10-week testing phase, Trial Promoter successfully generated and distributed 175 messages via Twitter and 350 messages via Facebook, a total of 525 messages. Figure 5 shows examples of messages that Trial Promoter generated automatically for distribution on social media.

### Correctness Analysis

The analysis of the test messages revealed that in a random sample of 25/525 messages the Trial Promoter algorithm used the correct message template without any text errors 100% of the time. However, we discovered a recurring issue in 13/525 messages (2.5%) where the algorithm used a question mark instead of an apostrophe (eg, “We’re looking for participants”). Furthermore, the analysis of the URLs showed that 525/525 messages (100%) included the correct URL. Finally, the disease hashtag analysis revealed that 24/525 distributed messages (4.6%) lacked the disease hashtag. Trial Promoter had not substituted the parameter “#disease” in the message template with the disease of the clinical trial in all cases. On average, Trial Promoter correctly used the message templates and substituted the message parameters (text, URLs, and disease hashtags) 97.7% of the time (1563/1600).

### Availability

The Trial Promoter software code is available under the MIT license [22,23]. Software code versions for technical and nontechnical users are accessible through the Trial Promoter website and hosted on GitHub [10]. To simplify the installation process for nontechnical users, we have written a script that deploys an instance of Trial Promoter to the public hosting service Heroku—within less than 30 minutes and without requiring technical knowledge of server setup and system administration. A fee of US $14 per month is required for hosting Trial Promoter on Heroku. Using the provided code, nontechnical users will be able to do the following: add clinical trial information for promotion—one trial at a time; import message templates—one at a time; automatically integrate with the social media management tool Buffer that automates the distribution of the messages; and create a dashboard that imports metrics from Buffer via a REST API, and from Twitter and Facebook via a CSV file. For technical users, detailed instructions for hosting Trial Promoter on an Ubuntu 14.04 LTS machine are also available on GitHub. Using the provided code, technical users will be able to do the following: import clinical trial information via a REST API—many trials at once; import message templates via a REST API—multiple templates at once; automatically integrate with Buffer that automates the distribution of the messages; and create a dashboard that automatically imports metrics from Buffer via a REST API, and that imports metrics from Twitter and Facebook via a CSV file. Finally, the full code used in the experiment described here is accessible through the Trial Promoter website and viewable on GitHub [10].

## Discussion

### Automated Clinical Trial Promotion

We have developed and tested Trial Promoter, a Web-based tool that automatically generates and distributes user-friendly social media messages about clinical trials. We chose the social media platforms Facebook and Twitter because members of disease communities frequently use them [2,24,25].

Trial Promoter managed to generate and distribute social media messages with a high level of correctness. We were able to improve the Trial Promoter algorithm with regard to the technical issues we encountered during testing. First, in 5% of the messages the algorithm had not substituted the generic disease hashtag parameter (#disease) with the corresponding disease hashtag taken from the clinical trial. We found that the substitution algorithm was both case and white-space sensitive (ie, # Disease, # disease, and #Disease). As a result, only the parameter #disease without a space and with lowercase “d” was replaced with the actual disease term that was associated with a clinical trial. We have modified the Trial Promoter algorithm, rendering the replacement of disease hashtags case and white-space insensitive, thereby resolving the issue for the future.

In light of millions of messages divulged by social media users on Twitter and Facebook every day, tailoring those messages to a specific audience is critical to cut through the noise. Hashtags provide a useful tool to target messages to specific topics and disease communities. The simple # symbol, known as a hashtag, for example, #leukemia, #rheum, is included in each message to indicate a topic, conversation, or event on Twitter [14] that the message relates to. Our ongoing work is focused on assessing the effectiveness of Trial Promoter for the promotion of clinical trial messages through a new Twitter account without followers. Following a Twitter user means to subscribe to a person’s feed, that is, stream of messages.

Second, in 2.5% of the messages the algorithm introduced a question mark instead of an apostrophe owing to encoding issues. This error was introduced while copying the message templates from a Google Docs file [24] into the code of our local Trial Promoter installation that we used during the testing phase. We consider this a rare technical issue specific to Google Docs where an apostrophe is encoded differently than text within a code editor. However, if necessary this type of technical issue can be addressed and corrected through the administrative dashboard of Trial Promoter.

**Figure 6:**
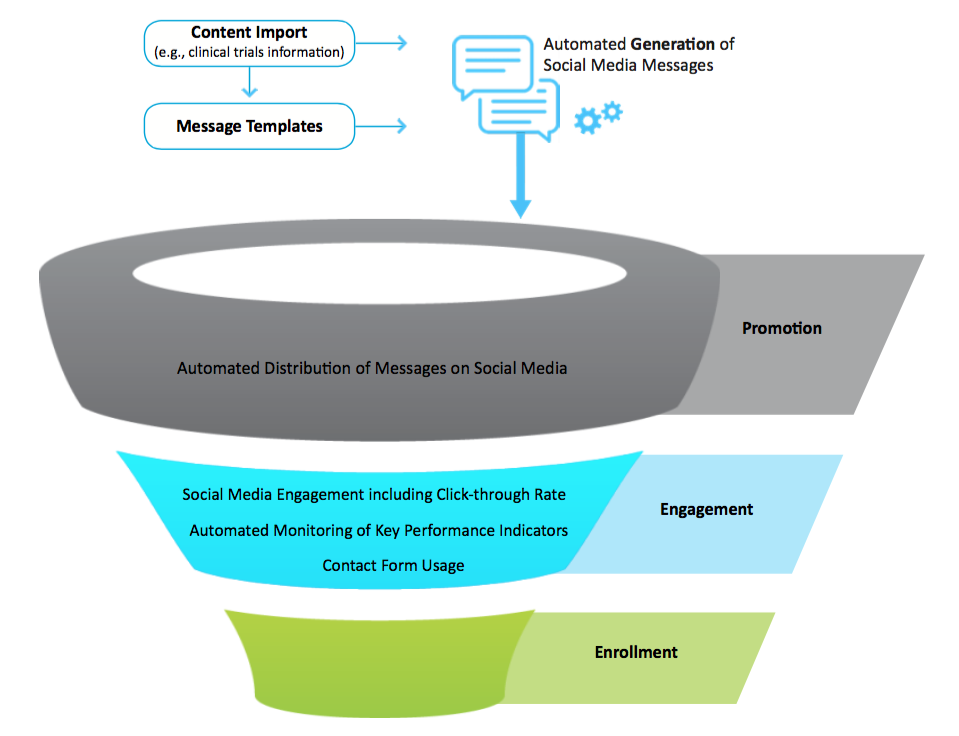
Trial Promoter is designed to facilitate two phases of the clinical trial recruitment process: the promotion (advertisement) and engagement phases.

The data thus far indicate that Trial Promoter serves as a promising tool to support clinical trial promotion via social media. More specifically, Trial Promoter is designed to facilitate two phases of the clinical trial recruitment process. Figure 6 illustrates these phases: the promotion (or advertisement) phase and the engagement phase. By publishing messages about clinical trials on social media, Trial Promoter supports the awareness-building phase of the clinical trial recruitment process. Several studies indicate that awareness changes attitudes toward clinical trials, enrollment, and the benefits of participation. More than 80% of patients were either unaware or unsure that participation in a clinical trial was an option at the time of diagnosis, and 75% of these patients said they would have been willing to enroll had they known it was possible [25,26]. These data indicate that improving the distribution of clinical trial information at limited cost may benefit clinical trial recruitment efforts.

Trial Promoter further generates opportunity for social media engagement by potential study participants, disease advocates, and others because social media is designed to facilitate interaction and conversation, for example, sharing, liking, following, and replying. The link engagement in the message that Trial Promoter generates and distributes is essential to triage visitors to the clinical trial landing page where they can find more information about the trial and potentially contact the study team using a compliant contact form.

### Saving Time and Cost

We wondered whether Trial Promoter could increase fiscal efficiencies of the clinical trial promotion. This is especially relevant in light of the high operational costs associated with clinical trials [4,27]. Nearly 30% of the time dedicated to clinical trials is spent on promotion, patient recruitment, and enrollment [28]. Despite this substantial amount of time and cost, more than 30% of all clinical trials fail to meet their enrollment targets, and more than 10% never enroll a single patient [29].

To test the theory as to whether Trial Promoter makes the promotion of clinical trials on social media more efficient fiscally, we compared the labor and cost of Trial Promoter with the labor and cost of a social media manager. During the 10-week testing phase, Trial Promoter automatically generated 525 user-friendly messages. The generation of a social media message takes an average of 5-15 minutes including selecting the content, writing the message, adding an optional image or video, and posting the message (based on internal analysis). According to jobs and recruiting site Glassdoor, the national average annual salary for a social media manager in 2015 was US $52,000 [30]. A social media manager would have required 43-131 hours of labor equivalent of US $1800-$5400 labor cost to generate the number of messages Trial Promoter has generated in 10 weeks (525). We argue that Trial Promoter helps to decrease the time and cost of labor required to generate and distribute information about clinical trials through social media. Although there were costs associated with the development of Trial Promoter (ie, personnel cost, server cost), those are minimal in comparison to the fact that the tool will be a free resource to the research community. It can be used repeatedly at no additional cost to investigators and research institutions. The tool may also help research institutions and investigators to gain efficiencies by streamlining and improving the clinical trials infrastructure and process so that those investigating new research questions could quickly draw on resources already in place instead of reinventing the wheel for each trial—in this case the broader dissemination of clinical trial information via social media.

Saving time and cost would not—in part—be possible without automation. However, the use of automated postings in digital marketing and social media is a controversial topic. We believe automation to serve patients, disease advocates, and research institutions alike when used appropriately and as long as the content is relevant and of value to the audience. Trial Promoter, for example, allows clinical study teams and others in charge of promoting clinical trials to design and write the posts at times when it is more convenient for them. Trial Promoter thereby not only saves them time, the tool also schedules and disseminates the social media posts at times when it is more convenient for the audience.

### Related Work

A number of studies have discussed the automation of the production of news and information. Automation offers new possibilities for creating content at scale, more quickly than a human could. So-called “bots,” that is, automated accounts on digital and social media (eg, Twitter, Facebook, Reddit, and Wikipedia) that distribute news and information, have been observed and studied in a variety of contexts: in social networks and human communication decisions [31,32], social shaping [33], content pollution [34], social metric gaming [35], ranking manipulation [36], infiltration [37], political astroturfing [38], recommendation [39], scholarship dissemination [40], activism or advocacy [41], and journalism [42]. Lokot and Diakopoulos concluded that news bots might enable innovation, such as niche and local news [42]. Different definitions have been introduced to describe these bots as “automated social actors”—software designed to act similarly to how humans might act in social spaces [31], as “software agents that interact on social networking services” [33], and as “automatic or semi-automatic computer programs that mimic humans and/or human behavior” [43]. However, future research will need to investigate how the public perceives news and information bots, whether they recognize bots as automated information services, if they are skeptical of content shared by a bot, and whether bots are ultimately effective in achieving the bottom line, for example, increase clinical study recruitment or foster the accessibility of public health information. Some small-scale work suggests that “while the software-generated content is perceived as descriptive and boring, it is also considered to be objective although not necessarily discernible from content written by journalists” [44]. Another study found that a Twitter bot sharing public health information was perceived as “credible,” “attractive,” and “competent,” suggesting that such “bots could be gainfully employed by [organizations] if properly harnessed” [45]. The authors are not aware of similar research that has tested the feasibility and effectiveness of automated postings of clinical research information. Yet the preliminary data in other fields look promising.

### Limitations

#### Limitations of the Study

The test trial we present here was focused on assessing the probability with which Trial Promoter generates and distributes correct messages about clinical trials. Future studies will be required to systematically assess the efficacy of Trial Promoter (or other algorithms) beyond fiscal efficiencies, determining the ability of machine-generated clinical trial information to foster the awareness of and engagement among target audiences such as patients, disease advocates, and physicians. Furthermore, it will be interesting to explore to what extent social media engagement with machine-generated content translates into increased clinical trial recruitment and enrollment rates.

#### Limitations of Trial Promoter

The current version of Trial Promoter does not automatically include images into the social media messages. Images, however, have been shown to be an important aspect of social media messages to increase engagement and information uptake [46]. The tool also does not automatically include mentions of influencers in the messages, that is, names of Twitter users with a lot of followers and reach—an important technique to increase the exposure of messages among target audiences. Additionally, Trial Promoter does not yet take into account awareness months when scheduling messages, for example, October is Breast Cancer Awareness Month. To increase the reach of the distributed information, Trial Promoter could increase the promotion of disease-related messages during awareness months.

### Conclusions

In summary, we present Trial Promoter and preliminary data indicating that the tool reliably automates the generation and distribution of correct clinical trial messages via social media. The Trial Promoter software code is freely available online. Although our local installation and pilot project focuses on clinical trials, Trial Promoter has the capability to support the generation and distribution of any type of content. Other examples of content include research news stories, peer-reviewed articles, and information about research experts and their expertise.

We hypothesize that machine-generated content helps research institutions and investigators to distribute clinical trial information more broadly and effectively. However, further studies around machine-generated content on social media will help to understand its role in facilitating patient engagement, increasing clinical trial awareness, and improving study recruitment and retention rates.

## Abbreviations

API: application programming interface
CSV: comma-separated values
HIV: human immunodeficiency virus
ID: identifier
KPI: key performance indicator
LTS: long-term support
REST: representational state transfer
USC: University of Southern California
UTM: Urchin Traffic Monitor
